# Socioeconomic factors associated with use of telehealth services in outpatient care settings during the COVID-19

**DOI:** 10.1186/s12913-024-10797-4

**Published:** 2024-04-09

**Authors:** Pearl C. Kim, Lo-Fu Tan, Jillian Kreston, Haniyeh Shariatmadari, Estella Sky Keyoung, Jay J. Shen, Bing-Long Wang

**Affiliations:** 1 Department of Health Care Administration and Policy, School of Public Health, University of Nevada in Las Vegas, Las Vegas, USA; 2 InnovAge PACE, San Bernardino, USA; 3 Optum Care, United Health Group, Las Vegas, USA; 4 Orange County School of the Arts, Santa Ana, USA; 5 Center for Health Disparities and Research, School of Public Health, University of Nevada in Las Vegas, Las Vegas, USA; 6 School of Health Policy and Management, Chinese Academy of Medical Sciences & Peking Union Medical College, Beijing, China

**Keywords:** Telehealth, Telemedicine, COVID-19, Socioeconomic disparities

## Abstract

**Background:**

To examine potential changes and socioeconomic disparities in utilization of telemedicine in non-urgent outpatient care in Nevada since the COVID-19 pandemic.

**Methods:**

This retrospective cross-sectional analysis of telemedicine used the first nine months of 2019 and 2020 electronic health record data from regular non-urgent outpatient care in a large healthcare provider in Nevada. The dependent variables were the use of telemedicine among all outpatient visits and using telemedicine more than once among those patients who did use telemedicine. The independent variables were race/ethnicity, insurance status, and language preference.

**Results:**

Telemedicine services increased from virtually zero (16 visits out of 237,997 visits) in 2019 to 10.8% (24,159 visits out of 222,750 visits) in 2020. Asians (odds ratio [OR] = 0.85; 95% confidence interval [CI] = 0.85,0.94) and Latinos/Hispanics (OR = 0.89; 95% CI = 0.85, 0.94) were less likely to use telehealth; Spanish-speaking patients (OR = 0.68; 95% CI = 0.63, 0.73) and other non-English-speaking patients (OR = 0.93; 95% CI = 0.88, 0.97) were less likely to use telehealth; and both Medicare (OR = 0.94; 95% CI = 0.89, 0.99) and Medicaid patients (OR = 0.91; 95% CI = 0.87, 0.97) were less likely to use telehealth than their privately insured counterparts. Patients treated in pediatric (OR = 0.76; 95% CI = 0.60, 0.96) and specialty care (OR = 0.67; 95% CI = 0.65, 0.70) were less likely to use telemedicine as compared with patients who were treated in adult medicine.

**Conclusions:**

Racial/ethnic and linguistic factors were significantly associated with the utilization of telemedicine in non-urgent outpatient care during COVID-19, with a dramatic increase in telemedicine utilization during the onset of the pandemic. Reducing barriers related to socioeconomic factors can be improved via policy and program interventions.

The Coronavirus disease 2019 (COVID-19) pandemic has significantly impacted the landscape of the health care delivery system, resulting in faster changes in traditional medical care infrastructure; there has been a transition from face-to-face healthcare services to more information and technology-based care through telemedicine. In order to reduce unnecessary exposure to COVID-19, mitigate the spread of the virus, and reduce surges in hospitals and clinics, the implementation of telemedicine as an alternative to in-person outpatient visits became a necessary component for non-emergency healthcare, with many hospitals choosing to discontinue in-person treatment [1, 2]. Doctors immediately started offering treatments via telehealth due to payment equality, the removal of administrative obstacles, and the Affordable Care Act of 1996 exemptions by the Department of Health and Human Services [3]. Additionally, almost all federal, state, and commercial insurers altered their telehealth coverages in reaction to the COVID-19 outbreak, which led to an increase in the usage of telehealth in actual practice. Increasing the population eligible for telehealth was one of many adjustments made as part of the expansion to make it easier for patients to get clinical treatment outside of in-person, face-to-face appointments [4].

Telehealth refers to the utilization of digital data and telecommunication services to help and encourage clinical health care, according to the Health Resources and Services Administration (HRSA) of the U.S. Department of Health and Human Services. The World Health Organization (WHO) defines telemedicine as the delivery of healthcare services by all healthcare professionals using information and communication technologies where distance is a critical factor [5, 6]. Although telehealth and telemedicine are used interchangeably, telemedicine refers to remote doctor-patient consultations that are services delivered by physicians only [7]. Remote diagnoses, e-prescriptions, e-consultations/specialist appointments, and digital transmissions of medical imaging are examples of telemedicine [7]. Technologies for telemedicine typically include videoconferencing, the internet, store-and-forward imaging, streaming media, and terrestrial and wireless communications.

Through video imaging and other technologies, telehealth allows medical practitioners to perform services remotely [8]. According to previous research, telehealth visits may provide patients with health outcomes that are equivalent to regular in-person doctor’s visits, with the added advantage of better access to treatment [9]. However, increases in telemedicine usage during the pandemic have raised concerns of exacerbating health disparities [10]. Individuals with insurance coverage, those who do not understand English, and elderly patients are less likely to utilize telehealth than in-person appointments [11–13]. Conversely, research from 2016 demonstrates that patients without health insurance had a 21% higher likelihood of favoring a telehealth consultation over a regular one [14]. Pediatric patients who did not speak English (1.7% vs. 2.7%) or who had Medicaid insurance (32.0 vs. 35.9%) had a lower likelihood of finishing a consultation via telemedicine [15]. Also, compared to those aged 31–45, individuals aged 46–60 were more likely to schedule telehealth meetings; compared to white patients, Black and other racially-identifying patients used telehealth less frequently [13, 16]. Another study identified that telehealth visits made up more than 50% of all visits, with Asian patients using telehealth less frequently than both Black and White patients. There was no difference in telehealth utilization among Black and White patients below 65 years old, however utilization among Asian and Hispanic patients was less than that of same-aged White patients [17].

Since the COVID-19 pandemic, there has been a large influx of literature regarding different aspects of telemedicine use. However, they had limited socioeconomic analyses, and very few studies used clinical data to examine the use of telemedicine in outpatient care during the pandemic. Therefore, the objective of this study was to examine potential changes in the utilization of telemedicine in non-urgent outpatient care in Nevada during the beginning of the COVID-19 pandemic and investigate potential factors associated with telemedicine use before and after the COVID-19 outbreak.

## Methods

This study was a retrospective secondary data analysis using the first nine months of 2019 and 2020 non-urgent outpatient visit data abstracted from the electronic health record (EHR) system of a large healthcare provider in the Southwest region of the United State. The EHR data was completely de-identified by the healthcare provider organization before being given to the research team for analysis to maintain anonymity of patients. A Data Use Agreement was approved by both the healthcare organization and the affiliated institution of the academic researchers in order to protect the privacy of individual health information through the Health Insurance Portability and Accountability Act (HIPAA). Further, the study was reviewed then exempted by the Institutional Review Board (IRB) of the University of Nevada, Las Vegas. The non-urgent care outpatient services include three departments: adult care, pediatric care, and specialty care. The utilization of telemedicine services in both years were compared. The 15 most frequent principal diagnoses were defined using the International Classification of Diseases, Tenth Revision, Clinical Modification for outpatient care prior and during the pandemic were also compared between 2019 and 2020.

Social demographic factors in association with use of telemedicine services were examined through multivariable analysis. The dependent variables were the use of telemedicine among all outpatient visits and using telemedicine more than once among those patients who did use telemedicine. The independent variables (i.e., sociocultural factors) included age (age groups-5 years old or younger, 6–17, 18–24, 25–34, 35–44, 45–54, 55–64, 65–74, >=75 years old), sex (female or male), race/ethnicity (White, Black, Hispanic/Latino, Asian/Pacific Islander, and other), primary spoken language (English, Spanish, and other), and health insurance program (Medicare, Medicaid, and private insurance). Given the small sample size, American Indians and Native Alaskans were grouped with the other group.

Multiple logistic regression was used to analyze factors associated with telemedicine use in 2020 only because there was virtually no telemedicine use in 2019. Clinical categories such as adult care, pediatric care, and specialty care (cardiology, gastroenterology, rheumatology, endocrinology, and neurology) were controlled in the analysis. Sensitivity analysis was conducted by analyzing the three clinical categories separately, and the results were fairly similar to those of analyzing all three together. It is important to note that EHR data in this study only includes audiovisual telemedicine visits.

## Results

**Fig. 1:**
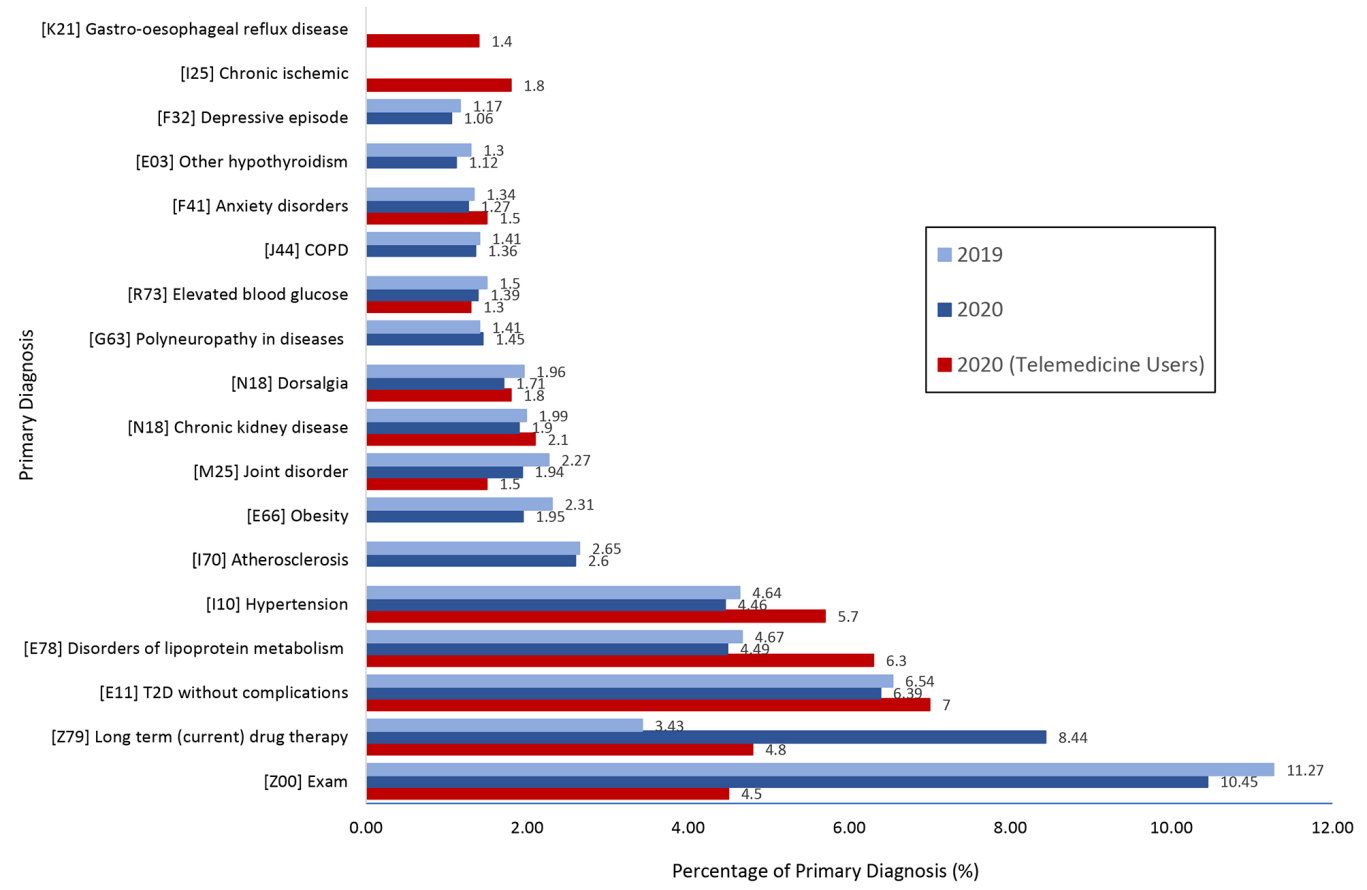
15 most frequent principal diagnosis of outpatient visits pre- and during the COVID-19 pandemic. *Notes*. [ ] indicates ICD-10 codes; COPD = chronic obstructive pulmonary disease; T2D = type 2 diabetics

There were 237,993 and 222,750 outpatient visits in the first nine months of 2019 and 2020, respectively, among which there were 16 and 24,228 telehealth visits in 2019 and 2020, respectively. The total number of patients who had 24,228 telehealth visits in 2020 was 19,503. Telemedicine services were virtually zero (16 visits out of 237,997 visits) in the first nine months of 2019 but increased to 10.8% (24,159 visits out of 222,750 visits) in the nine months of 2020. Figure 1 depicts the most frequent principal diagnoses of outpatient visits prior to and during the COVID-19 pandemic. The top 15 most frequent diagnoses for the all-patient visits were similar between the two years while the volume of long-term (current) drug therapy increased by 158% (21,907 in 2019 to 56,611 in 2020). The five most frequent primary diagnoses for all patients in 2019 and 2020 were exam, long term drug therapy, type 2 diabetes without complications, disorders of lipoprotein metabolism, and hypertension. Specifically for telemedicine users in 2020, type 2 diabetics, disorders of lipoprotein metabolism, and hypertension were the top three primary diagnoses, showing consistencies between telemedicine and in-person diagnoses (Fig. 1). Characteristics of telemedicine users in outpatient care during the COVID-19 period in 2020 are shown in Table 1. The total number of patients who used telemedicine in 2020 was 19,503 (Table 1). Among those 19,503 patients who used telemedicine in 2020, over 60% were females, about half were minorities, about 13% spoke languages other than English, 42.7% were covered by Medicare, and 16.0% were covered by Medicaid. In addition, about two-thirds were treated in adult medicine, and 18.1% had more than one telemedicine visit (Table 1).

**Table 1:** Characteristics of users of telemedicine in outpatient care during the Covid-19 period, 2020 (*N* = 19,503)

	Frequency	Percent
Gender		
Female	11,789	60.5%
Male	7,713	39.5%
Race		
Asian	1283	6.6%
Black	2969	15.2%
White	9,785	50.2%
Other	2,298	11.8%
Unknown	3,168	16.2%
Ethnicity		
Hispanic/Latino	2,650	13.6%
Non-Hispanic/Latino	8,846	45.4%
Unknown	8,007	41.1%
Language		
English	16,958	87.0%
Spanish	894	4.6%
Other	1,428	8.4%
Insurance		
Private	8,038	41.2%
Medicare	8,320	42.7%
Medicaid	3,125	16.0%
Multiple telehealth visits		
Yes	3,250	18.1%
No	15,983	82.0%
Medical Specialty		
Adult Medicine	12.935	66.3%
Specialty		24.4%
Cardiology	1,710	8.8%
Gastroenterology	1,531	7.0%
Rheumatology	709	3.6%
Endocrinology	695	3.6%
Neurology	281	1.4%
Pediatric	1,174	6.0%
Primary Medicine	468	2.4%

Table 2 describes factors associated with the utilization of telemedicine services among all outpatient visits during the COVID-19 period. Among all outpatient visits in 2020, older and younger age groups were less likely to use telemedicine compared to the median age group 35–44 (aOR = 0.74, CI=[0.68–0.81] for 18–24, aOR = 0.85, CI=[0.80–0.89] for 55–64 ages, aOR = 0.71, CI=[0.67–0.76] for 65–74 ages, and aOR = 0.69, CI=[0.65–0.74] for older than 75 years old; males, as compared to females, were less likely to use telemedicine (adjusted odds ratio (aOR) = 0.86, 95% confidence interval (CI) = [0.83–0.88]); Asians and Latinos/Hispanics, as compared to Whites, were less likely to use telehealth (aOR = 0.85, CI = [0.81–0.90] for Asians, and aOR = 0.89, CI = [0.85–0.94] for Latinos/Hispanics); Spanish-speaking patients and other non-English speaking patients, as compared with English-speaking patients, were less likely to use telehealth (aOR = 0.68, CI = [0.63–0.73] for Spanish speaking patients and aOR = 0.93, CI = [0.88–0.97] for other language speaking patients); and both Medicare and Medicaid patients were less likely to use telehealth than their privately insured counterparts (aOR = 0.94, CI = [0.89–0.99] for Medicare patients and aOR = 0.91, CI = [0.87–0.97] for Medicaid patients). In addition, patients treated in pediatric and specialty care were less likely to use telemedicine (aOR = 0.76, CI=[0.60–0.96] for Pediatric, and aOR = 0.67, CI=[0.65–0.70] for Specialty), as compared with patients who were treated in adult medicine.

**Table 2:** Factors associated with use of telemedicine services among all outpatient visits during the Covid-19 period, 2020 (*N* = 222,750)

Independent Variables	Odds Ratio	95% CI	p-Value
Age Group (in year)			
<= 5	0.27	[0.21–0.35]	< 0.001
6–17	0.49	[0.38–0.63]	< 0.001
18–24	0.74	[0.68–0.81]	< 0.001
25–34	0.97	[0.91–1.03]	0.309
35–44 (reference)			
45–54	0.92	[0.87–0.97]	0.002
55–64	0.85	[0.80–0.89]	< 0.001
65–74	0.71	[0.67–0.76]	< 0.001
>= 75	0.69	[0.65–0.74]	< 0.001
Gender			
Male	0.86	[0.83–0.88]	< 0.001
Female (reference)	1.00		
Race/Ethnicity			
Asian	0.85	[0.81–0.90]	< 0.001
Black	1.02	[0.98–1.07]	0.281
Hispanic/Latino	0.89	[0.85–0.94]	< 0.001
Other	0.93	[0.88–0.99]	0.026
White (reference)	1.00		
Language			
Spanish	0.68	[0.63–0.73]	< 0.001
Other	0.93	[0.88–0.97]	0.002
English (reference)	1.00		
Insurance			
Medicare	0.94	[0.89–0.99]	0.011
Medicaid	0.91	[0.87–0.94]	< 0.001
Private (reference)	1.00		
Medical Specialty			
Pediatric	0.76	[0.60–0.96]	0.023
Specialty*	0.67	[0.65–0.70]	< 0.001
Adult Medicine (reference)	1.00		

*Note*: *includes cardiology, gastroenterology, rheumatology, endocrinology, and neurology

Furthermore, factors associated with frequent telemedicine users were shown in Table 3. Among patients who did use telehealth services, males, as compared to females, were less likely to have multiple telehealth visits (aOR = 0.89, CI = [0.82–0.96]); Asians, as compared to their white counterparts, were less likely to have multiple telehealth visits (aOR = 0.85, CI = [0.81–0.90]); patients speaking languages other than English or Spanish, as compared with their English-speaking counterparts, were less likely to have multiple telehealth outpatient visits (aOR = 0.86, CI = [0.74–0.99]); whereas both Medicare and Medicaid patients were more likely to have multiple telehealth visits than patients covered by private health insurance (aOR = 1.33, CI = [1.16–1.52] for Medicare patients and aOR = 1.19, CI = [1.07–1.34] for Medicaid patients).

**Table 3:** Factors associated with frequent telemedicine users in outpatient care during the Covid-19 among telehealth users, 2020 (*N* = 19,503)

Independent Variables	Odds Ratio	95% CI	p-Value
Age Group (in year)			
<= 5	0.80	[0.33–1.92]	0.612
6–17	0.63	[0.27–1.52]	0.305
18–24	0.61	[0.43–0.87]	0.006
25–34	0.90	[0.72–1.11]	0.308
35–44 (reference)			
45–54	1.01	[0.86–1.18]	0.947
55–64	1.07	[0.92–1.25]	0.364
65–74	0.98	[0.82–1.18]	0.859
>= 75	1.06	[0.87–1.29]	0.559
Gender			
Male	0.89	[0.82–0.96]	0.002
Female (reference)	1.00		
Race/Ethnicity			
Asian	0.83	[0.71–0.98]	0.024
Black	1.06	[0.95–1.17]	0.317
Hispanic/Latino	1.04	[0.92–1.19]	0.540
Other	0.92	[0.76–0.90]	0.344
White (reference)	1.00		
Language			
Spanish	0.87	[0.71–1.08]	0.201
Other	0.86	[0.734–0.99]	0.041
English (reference)	1.00		
Insurance			
Medicare	1.33	[1.16–1.52]	< 0.001
Medicaid	1.19	[1.07–1.34]	0.002
Private (reference)	1.00		
Medical Specialty			
Pediatric	1.12	[0.48–2.57]	0.797
Specialty*	0.93	[0.86–1.01]	0.105
Adult Medicine (reference)	1.00		

*Note*: *includes cardiology, gastroenterology, rheumatology, endocrinology, and neurology

## Discussion

This cross-sectional analysis of telemedicine use during the emergence of the COVID-19 pandemic (January– September 2020) demonstrated dramatic increases in telemedicine utilization in non-urgent outpatient care compared to the pre-pandemic period (January-September 2019). Contributing factors that could have affected telemedicine use during the pandemic include the following policy adaptations and guidance regulations. In February 2020, the Centers for Disease Control and Prevention (CDC) issued guidelines for social distancing practices and recommended that healthcare facilities and providers offer clinical services through virtual care in order to mitigate the spread of the virus and reduce hospital case surges. The increased number of telemedicine utilization during COVID-19 might also be related to the policy changes and regulatory waivers from the CDC in response to COVID-19 and provisions of the U.S. Coronavirus Aid, Relief and Economic Security Act on March 6, 2020. Under the emergency policies and waivers, no preexisting patient-provider relationship is required, virtual visits from the patient’s home or audio-only are allowed, and reimbursements for telemedicine improved dramatically through the expansion of Medicare’s telehealth coverage [6].

During the pandemic, the increase of telemedicine utilization diverged significantly across clinical specialties, medical conditions, and patient demographics. Delays in medical care were observed in the U.S. with concerns of possible COVID-19 exposure and limiting non-essential care; The CDC reported that about 41% of U.S. adults experienced delayed medical care in 2020 [18]. Similarly, the scope of deferred care during the pandemic was shown in our study. We observed that the number of visits for the fifteen most frequent principal diagnoses in outpatient care remained similar between the pre- and during the pandemic period, however, the volume of long-term (or current) drug therapy doubled from 2019 to 2020. Our findings indicate that telemedicine may have provided the opportunity to dramatically increase drug therapy and thereby defer patient care during the pandemic. Studies showed that routine and chronic care management were the most reported types of deferred care [19, 20], yet patients with common chronic conditions showed lower use of telemedicine during the pandemic [10]. Contrary to previous studies, our study indicated that the scope of outpatient care for common chronic conditions such as hypertension and diabetes did not alter with the use of telemedicine during the pandemic.

Telemedicine use also varied across socioeconomic characteristics of patients. Our study demonstrates that inequities exist in regards to utilization of outpatient care via telemedicine for socioeconomically diverse patients during the pandemic. We found that younger and older age, Asian and Hispanic/Latino race, Spanish-speaking patients, and patients with Medicaid insurance were independently associated with less utilizations of telemedicine. Our findings are consistent with prior research regarding telemedicine use in overall and primary care [10, 13, 19, 21, 22].

As older adults tend to have slower rates of technology adoption, less experience with technology, and low digital health literacy, a common concern has arisen regarding their access to telemedicine and virtual care [13, 23–25]. Our findings highlight this disparity in telemedicine access as older adults are less likely to utilize telemedicine than their younger counterparts. Notably, patients aged 55 to 64, 65 to 74, and 75 and older were found to be 15%, 29%, and 31% less likely to access telemedicine in comparison to adults aged 35 to 44. More studies must be done to identify potential barriers to telemedicine access in young people; a potential hypothesis is that younger patients may have different perceptions of their medical needs, potentially attributing to less telemedicine usage. Additionally, our study discovered that males had less telemedicine use compared to females during the pandemic, [13, 26]. This is consistent with past studies that indicated that females tend to have more medical visits and prefer telemedicine care over in-person care [26].

Furthermore, our study demonstrated that socioeconomic disparities in accessing telemedicine care were present during the pandemic. Both Asians and Hispanics/Latinos were less likely to use telemedicine compared to Whites. While Asians have been known to have high technology adoption rates and broadband service use, sociocultural factors may have played a role in our findings [27]. Asian and Hispanic/Latino patients may experience specific language and cultural barriers, which may explain less telemedicine use as they may prefer to stay with their providers in person in order to retain communication and familiarity. While their providers might have conducted some telemedicine visits, follow-up visits could be done by mid-level providers, leading to patient-doctor relationship discontinuities for the patient. A recent study in California identified language as an essential barrier to telehealth use as primary care and specialty ambulatory clinics have showed that non-English speaking patients were less likely utilize telemedicine [13, 28]. Our study also showed language as an essential barrier to telemedicine use in outpatient care, as non-English speaking patients were less likely to use telemedicine. Compared with in-person visits, effective communications were a common challenge during telemedicine [29]. Barriers to speaking up and asking questions and establishing a provider-patient relationship via telemedicine can create challenges for non-English speaking patients. Federal laws require Medicare and Medicaid to provide translation services to patients in their preferred language, however, not all providers offer language services [28]. The telemedicine platform of the healthcare provider in our study provided the use of a third-party vendor that could be contacted for translation services during live telemedicine visits. However, even with these accommodations, our study showed that telemedicine was still challenging for patients with limited English proficiency, making them less likely to repeatedly use telemedicine. The provision of linguistically appropriate care in telemedicine will not only help reduce language disparities but also eliminate other systematic and cultural barriers for patients with limited English proficiency.

There are three main different types of healthcare payers in the United States. Government payers include U.S. government-funded health insurance plans such as Medicare and Medicaid. Medicare is federal health insurance for anyone age 65 and older or people with certain disabilities and conditions. Medicaid is a state and federal program that provides health coverage to people with limited income and resources. Most individual and group health insurance plans are covered by private payers which include non-insurance payment such as paying cash directly. Our study defines private insurance as both commercial and private payers. Our study found that patients with government-funded insurance payers, both Medicare and Medicaid patients, were less likely to use telemedicine. However, for patients that did use telemedicine, they tended to use it multiple times. Past studies have shown mixed findings regarding telehealth use and payer status. Previous studies have shown that there is an association between patients with Medicaid and Medicare with lower healthcare utilization and telemedicine [13]. A Missouri study showed that Medicaid and Medicare patients had relatively higher odds of telehealth use compared to patients with private insurance during the COVID-19 telehealth expansion [30]. Findings from our study may reflect the early implementation of telemedicine at our healthcare system. Our health system has provided telemedicine in urgent care for private insurance patients since 2014, Medicaid patients since 2016, and Medicare patients since 2018. Therefore, patients with private insurance were more familiar with telemedicine than patients with government payers, potentially leading to higher telemedicine visits for those patients during the pandemic. While prior to the pandemic telemedicine was only used for urgent care, familiarity with the technology may have led to increases in non-urgent outpatient visits. Despite having less telemedicine use, patients with government-funded health insurance who had telemedicine visits tend to utilize telemedicine more frequently. A potential explanation for this finding is the advantages of telemedicine, as it reduces transportation costs, is convenient, and provides quality care to the patient with mobility limitations.

Our study also showed medical specialties as a potential contributing factor associated with telemedicine use during the pandemic. Patients in pediatric and specialty care were less likely to use telemedicine compared to patients in adult medicine. This may have been due to patient concerns regarding quality of care; provider preference of providing telemedicine care may have differed depending on their medical specialties in addition to the needs of in-person care [31]. However, reasons for this preference among physicians are unclear and may suggest an area for further research in order to identify specific barriers.

We acknowledge that our study had limitations. First, the data in this study was from a sample of large outpatient care providers in a single state, meaning that our findings may not be generalizable across all virtual encounters conducted during the study period. Second, our study defined the first nine months of 2020 as a post-COVID-19 period without considering any COVID-19-related policy implementations such as Medicare’s telehealth coverage expansion on March 17, 2020 and the healthcare system shutdown in Nevada on March 15, 2020 due to nationwide guidelines. Therefore, our findings might underestimate the impact of the COVID-19 pandemic on telemedicine use. Further, studies of telemedicine utilization beyond 2020 will help us to understand impact of post-pandemic on socioeconomic factors associated with telehealth use.

## Conclusions

Telemedicine utilization has increased dramatically since the pandemic, assisted by technological advancement and availability. Nevertheless, disparities exist for different races/ethnicities and non-English speakers. In order to reduce barriers related to socioeconomic factors, policy and program interventions must be improved in order to meet the new healthcare demands set in place by the transformation of healthcare delivery models. For example, enhancing language-related communication supports can likely reduce disparities in patient telehealth use for those with varied sociocultural backgrounds and socioeconomic statuses.

## Data Availability

Data will be available based on request. Please contact Jillian Kreston at jillian.kreston@optum.com.
